# Integrated traditional Chinese and conventional medicine in treatment of severe community-acquired pneumonia: study protocol for a randomized placebo-controlled trial

**DOI:** 10.1186/s13063-018-3005-9

**Published:** 2018-11-12

**Authors:** Haifeng Wang, Jiansheng Li, Xueqing Yu, Su-yun Li

**Affiliations:** 1 Collaborative Innovation Center for Respiratory Disease Diagnosis and Treatment and Chinese Medicine Development of Henan Province, Henan University of Traditional Chinese Medicine, Zhengzhou, People’s Republic of China; 2 Department of Respiration, The First Affiliated Hospital of Henan University of Traditional Chinese Medicine, Zhengzhou, People’s Republic of China; 3 The Geriatric Department, Henan University of Traditional Chinese Medicine, Longzihu University Town, Zhengdong New District, Zhengzhou, 450046 People’s Republic of China

**Keywords:** Community-acquired pneumonia, Pulmonary infection, Traditional Chinese medicine, Treatment, Clinical trials

## Abstract

**Background:**

Community-acquired pneumonia, especially severe community-acquired pneumonia (SCAP), remains the leading cause of death in the world. Despite advances in antimicrobial therapy, the mortality rates due to SCAP have not decreased significantly since antibiotics became routinely available. Traditional Chinese medicine (TCM) has been used for treating pneumonia for thousands of years. It is popular and widely practiced in Asia. In recent decades, evidence from both clinicians and patients suggests that TCM has some beneficial effect on SCAP. Thus, this study aims to compare the efficacy of a combination of a conventional drug and TCM to the conventional drug alone, to provide a scientific basis for clinical decisions.

**Methods/design:**

A prospective, multi-center, single-blinded, double-dummy, and randomized controlled clinical trial is being conducted to test the therapeutic effects of a combination of conventional medicine and TCM versus conventional medicine in the treatment of SCAP. A total of 198 patients will be enrolled in this study, with 99 in each treatment group (combination group or conventional medicine group). The TCM will be administered twice daily for 28 days. All patients will be followed for 3 months. The primary outcome measure is treatment failure, which is defined as clinical deterioration. Secondary outcome measures are time to clinical stability, length of hospital stay, in-hospital mortality, SOFA questionnaire, quality of life and cost of treatment.

**Discussion:**

It is hypothesized that the combination of a conventional drug and TCM will reduce treatment failure, time to clinical stability, length hospital of stays, and in-hospital mortality, and improve the quality of life of SCAP patients.

**Trial registration:**

ClinicalTrials.gov, NCT03185923. Registered on 20 June 2017.

**Electronic supplementary material:**

The online version of this article (10.1186/s13063-018-3005-9) contains supplementary material, which is available to authorized users.

## Background

Community-acquired pneumonia (CAP) is the leading cause of death due to infection worldwide [1, 2]. Despite advances in antibiotic treatment, mortality among hospitalized patients with CAP is still high, especially in those with severe pneumonia [3] and in those who experience treatment failure [4]. Patients with treatment failure, which is defined as clinical deterioration, may have higher rates of mortality compared with those who do not experience treatment failure [4], so it is possible to use treatment failure as a surrogate parameter for mortality. In patients with CAP, an excessive host inflammatory response is associated with treatment failure during intensive care unit admission and with mortality [5, 6].

Many different organizations have worked to improve the care of patients with CAP. Such efforts are warranted, because CAP, especially severe community-acquired pneumonia (SCAP), remains the leading cause of death globally. Despite advances in antimicrobial therapy, mortality rates due to SCAP have not decreased significantly since penicillin became routinely available.

Traditional Chinese medicine (TCM) has been used for thousands of years for treating pneumonia. It is popular and widely practiced in many countries around the world. In recent decades, evidence from both clinicians and patients suggests that TCM has some beneficial effect on SCAP [7, 8].

At present, many complementary therapies are offered for patients with SCAP. Moreover, it is not very clear whether TCM is a suitable therapy for SCAP. Thus, this study aims to compare the efficacy of a combination of a conventional drug and TCM to the conventional drug alone, to provide a basis for clinical decisions.

This multi-center randomized controlled trial will compare the efficacy of two therapies for patients with SCAP. In total, 198 subjects will be randomly assigned to one of the therapies (conventional drug alone or the combination of the conventional drug and TCM) for 28 days of treatment. After the treatment period, the subjects both two arms will be followed up for 3 months. The primary outcome will be treatment failure. The secondary outcomes are time to clinical stability, length of hospital stay, in-hospital mortality, sequential organ failure assessment (SOFA), quality of life (CAP-PRO), and health economics.

## Methods/design

### Objective and design

This is a multicenter, pragmatic, randomized, controlled trial to evaluate the effectiveness of integrated TCM treatment for SCAP patients. A total of 198 patients will be recruited and randomly assigned to one of the two treatment groups. After the 28 days of treatment, they will be followed for another 3 months. The primary outcome (treatment failure) and secondary outcomes (time to clinical stability, length hospital of stay, in-hospital mortality, SOFA questionnaire, quality of life, and health economics) will be assessed at different points within the trial.

The study has been approved after meeting conditions imposed by the ethical research committee of the First Affiliated Hospital of Henan University of Traditional Chinese Medicine (identifier 2017HL-002-01). This trial adheres to the Declaration of Helsinki and was registered at ClinicalTrials.gov (NCT03185923) on 20 June 2017. The protocol follows the recommendations of the SPIRIT initiative (Additional file 1) [9], and the trial results will be reported according to the latest version of the CONSORT statement [10].

### Study setting and participants

All adult patients with SCAP at respiratory departments in 11 tertiary-care hospitals will be screened and enrolled. Eligible patients will be asked for their informed consent and then centrally randomized to either the integrated traditional Chinese and conventional medicine group or to the conventional medicine group in a ratio of 1:1. All medications will be taken orally for 28 days. If the patient is not able to give informed consent at the time of hospitalization due to the severity of their CAP, informed consent from their next of kin and an independent physician will be obtained. In these cases, informed consent from the patient will be obtained as soon as possible. Patient recruitment started in July 2017 and is planned to be completed in December 2019.

To be included, patients must be aged between 18 years and 80 years, and must meet the criteria for SCAP (defined with the modified American Thoracic Society criteria) [11] and the criteria for TCM syndromes. TCM syndrome differentiation relates to pathological changes: phlegm-heat obstruction in the lung, pulmonary stagnation of phlegm, pathogenic heat trapped in the pericardium, or pathogenic depression.

The following patients are excluded:

- Pregnant and lactating women
- Patients with trauma, hematologic malignancies, various solid tumors, obstetric complications, aspiration pneumonia, fungal pneumonia, HIV-related pneumocystis carinii pneumonia, tuberculosis, bronchiectasis with infection and a pulmonary abscess, dementia, mental illness, neuromuscular disorders affecting the respiratory motor function, severe cardiovascular disease, severe liver disease, or kidney disease
- Patients who are unwilling to cooperate
- Patients who were discharged from hospital within 2 days or require an operation
- Patients with severe immunosuppression (due to HIV infection, immunosuppressive conditions, or medication).
- Patients who have participated in other clinical studies in the past 4 weeks
- Patients unwilling to sign the informed consent.

### Randomization

**Fig. 1:**
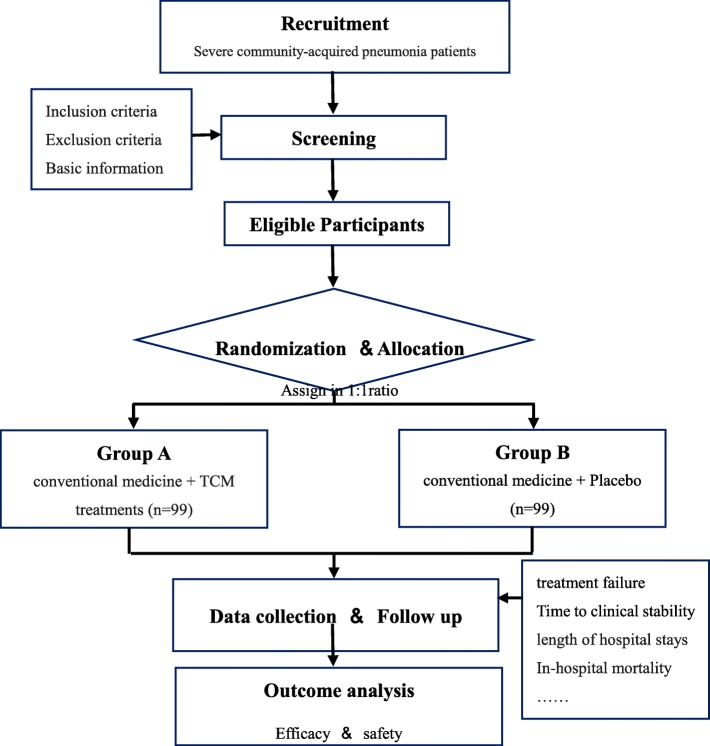
Flow chart of study design. TCM traditional Chinese medicine

Participants who meet the inclusion criteria and sign the informed consent form will be allocated to a group (Fig. 1). A stratified and block randomization design has been adopted. There are two groups and the distribution ratio is 1:1. The block size is six. Patients are stratified at the time of study entry by study center. Allocation is based on random numbers from 1 to 198 generated by SAS 9.2 and saved in a sealed envelope by an independent clinical statistician. In a clinical emergency, the individual’s randomization code and group allocation can be quickly identified by checking the envelope. The randomization design is provided by the Jiangsu Famous Medical Technology Co., Ltd.

### Study protocol

All patients are being treated according to current CAP guidelines [11]. Antibiotic therapy guided by an established procalcitonin algorithm is encouraged. The time of discharge is not determined by the study investigators, but chosen by the treating physician team.

For the combination of conventional medicine and TCM, patients will be given herbal interventions based on TCM syndrome patterns: *qing-fei jie-du hua-tan* granules (Sanjiu Medical & Pharmaceutical Co., Ltd.) for phlegm-heat obstruction in the lung, *zao-shi hua-tan xie-fei* granules for pulmonary stagnation of phlegm, *qing-xin kai-qiao* granules for pathogenic heat trapped in the pericardium, *shenmai* injections for yin exhausted syndrome, and *shenfu* injections for yang exhausted syndrome. The herbal interventions are recognized as a holistic intervention. The TCM granules are compound preparations of Chinese herbs and the main components are listed in Table 1. Each type of granule comes in bags, which are produced and packed by Sanjiu Medical & Pharmaceutical Co., Ltd. Test results of drug quality were consistent with the required quality standards. Each type of granule will be given orally, two bags each time, twice a day for 28 days.

**Table 1:** Main components of traditional Chinese medicine

Chinese name	Latin name	Amount (g)
*Qing-fei jie-du hua-tan* granule for phlegm-heat obstruction in the lung		
*Huang qin*	Radix scutellariae baicalensis	9
*Zhe bei mu*	Bulbus fritillariae thunbergii	9
*Gua lou*	Fructus et semen trichosanthis kirilowii	20
*Xi yang shen*	Radix panacis quinquefolii	6
*Zao-shi hua-tan xie-fei* granule for pulmonary stagnation of phlegm		
*Fa ban xia*	Prepared pinellia tuber	12
*Hou pu*	Cortex magnoliae officinalis	9
*Fu ling*	Poria	15
*Zi su zi*	Fructus perillae argutae	9
*Qing-xin kai-qiao* granule for pathogenic heat trapped i the pericardium		
*Shui niu jiao*	Cornu bubali	30
*Sheng di huang*	Radix rehmanniae	15
*Xuan shen*	Radix scrophulariae	12
*Mai dong*	Radix ophiopogonis japonici	12

For the conventional medicine group, patients will be given placebo herbal interventions according to the TCM syndrome.

### Data collection

Baseline data are collected by electronic case report forms compliant with good clinical practice. They include date and time of randomization, date of birth, sex, and medical history (dyspnea, cough, smoking history in pack-years, current smoking status in packs per day, relevant comorbidities, degree of autonomy at home, clinical items of pneumonia, pneumonia severity index, extent of pneumonia, and presence of underlying chronic obstructive pulmonary disease as assessed by patient history and medical documents).

In both groups, hospitalized patients are reassessed clinically every 24 h (including vital signs). Details of doses of all prescribed antimicrobials during the study period are recorded. In hospitalized patients, hospital discharge is recommended if oral intake is feasible, vital signs have been stable for ≥24 h, and there is no evidence of an acute serious comorbidity that necessitates hospitalization. On days 1, 4, 7, 14, and 28 the patient will be asked to complete a short questionnaire on their respiratory symptoms.

### Follow-up

**Fig. 2:**
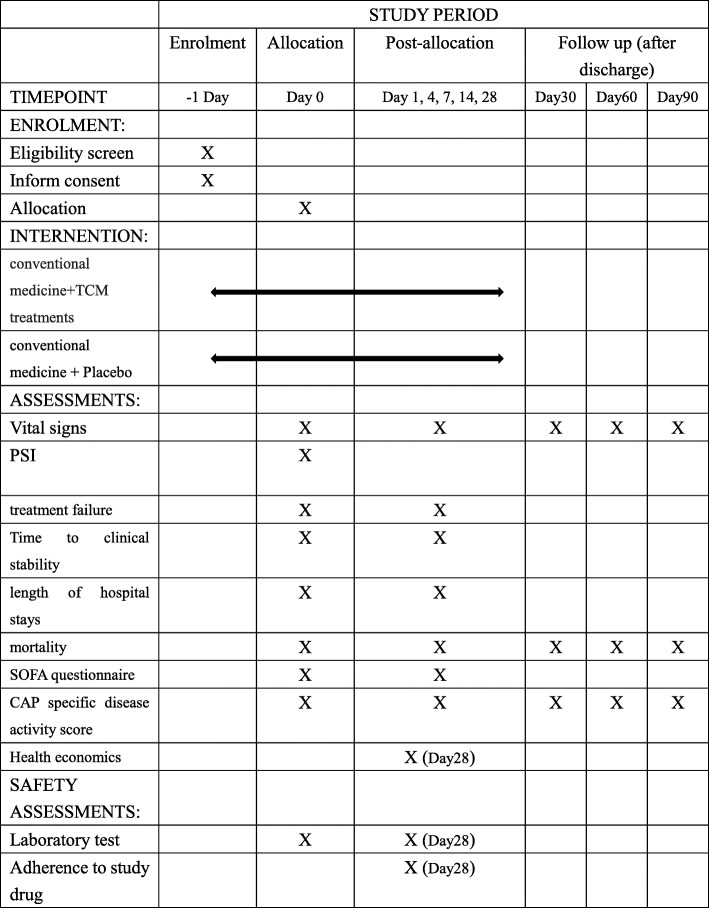
Schedule of enrolment, interventions and assessments (as per SPIRIT [9]). CAP community-acquired pneumonia, PSI, pneumonia severity index, TCM traditional Chinese medicine

Structured follow-up telephone interviews for secondary outcomes after discharge are performed on days 30, 60, and 90 (Fig. 2). If the patient cannot be contacted or cannot give sufficient information, their primary-care physician will be interviewed. Follow-up items include assessment of adverse events (such as infections, recurrent pneumonia, and re-hospitalization), and the standardized questionnaire on respiratory symptoms.

### Outcome measures

#### Primary outcome measure

The primary endpoint is treatment failure, which includes treatment failure that occurred early or late. Early treatment failure is defined as clinical deterioration within 72 h of treatment (including shock, need for invasive mechanical ventilation not present at baseline, or death). Late treatment failure is defined as radiographic progression (increase of ≥50% of pulmonary infiltrates compared with baseline), persistence of severe respiratory failure (ratio of PaO_2_ to fraction of inspired oxygen < 200 mmHg with respiratory rate ≥ 30 breaths/min in patients not intubated), need for invasive mechanical ventilation not present at baseline, or death 72 h after treatment initiation. These criteria have been used previously with modifications [12].

#### Secondary outcome measures

We will consider the following secondary endpoints.

##### Time to clinical stability

Clinical stability occurs when [11]: temperature ≤37.2 °C, heart rate ≤100 beats/min, systolic blood pressure ≥90 mmHg, and arterial oxygen tension ≥60 mmHg if the patient is not receiving supplemental oxygen. In patients receiving oxygen therapy at home, stability is achieved when their oxygen needs were the same as before admission.

##### Length of hospital stay

The time to the earliest possible hospital discharge (days) is based on objective criteria (able to take food, liquids, and drugs orally; vital signs stable for > 24 h; recovered from CAP-related worsening of mental status; and no evidence of an acute serious CAP-related comorbidity that necessitates hospitalization). This endpoint will minimize any potential bias due to extended hospitalization for non-disease-specific reasons (a request from the patient or their relatives, lack of adequate home care, inability to transfer to a nursing home, or lack of assurance about compliance with treatment) [13].

##### In-hospital mortality

All-cause mortality will be recorded after 28 treatment days and at 3 months during the follow-up period.

##### SOFA questionnaire

The clinical symptom questionnaire for SCAP will be assessed at baseline, and at days 0, 4, 7, 14, and 28 of the treatment phase.

##### Community acquired pneumonia-clinician report outcome (CAP –CRO)

A CAP-specific disease activity score, daily function, and health state will be assessed at baseline, at days 4, 7, 14, 21, and 28, and at the follow-ups at 30, 60, and 90 days.

##### Health economics

The cost of the treatment phase will be recorded for the 28 days.

#### Adverse event reporting

Side effects and adverse effects of TCM treatment will be assessed with a questionnaire. Any adverse events will be listed in the trial record and followed up to completion by the trial coordinators and respiratory physicians. Adverse events will be scored using a six-point scale: 0 = none, 1 = minimal, 2 = mild, 3 = moderate, 4 = severe, and 5 = extremely severe. Participants can report adverse events at any time throughout the trial and will receive advice accordingly. Serious adverse events will be reported to the reviewing human research ethics committee and the site ethics committees within the timeframe specified by the lead ethics committee. Throughout the study period trial, coordinators will contact the participant’s usual treating doctor to record relevant data on presentations.

Participants may withdraw from the study for any reason at any time without repercussions. They will be withdrawn by investigators only if it is deemed medically unsafe for them to continue. Dropouts will not be replaced.

#### Sample size considerations

This study is designed to show the superiority of the TCM group compared to placebo treatment. Our primary hypothesis is that adjunct TCM treatment in patients with SCAP will result in reduced treatment failure compared to the placebo. To estimate the frequency of our primary endpoint, we used data for SCAP patients from completed studies [12]. Based on these previous trials, we assumed a treatment failure rate of 30% in the placebo group and 10% in the adjunct TCM group over 28 days. Thus, a sample size of 198 patients is needed to achieve a statistical power of 90% (two-sided type-1 error of 5% in both scenarios). We intend to recruit 198 study patients from July 2017 to December 2019.

#### Intention-to-treat and per-protocol population

Following the intention-to-treat principle, all patients receiving at least one dose of study medication will be included in the analysis with group allocation as randomized. Patients found to have violated the inclusion criteria or met the exclusion criteria due to information that was not available at study entry will be excluded post randomization. Criteria for excluding patients from the per-protocol analysis are, in addition, withdrawal of informed consent or non-compliance with the study medication or the concomitant procedures. Therefore, patients stopping or pausing the study medication at any time will not be considered in the per-protocol analysis. A patient will not be followed up for any study measure if they withdraw informed consent. In all other cases, the follow-up will be performed as planned.

#### Statistical analysis

A descriptive statistical analysis will be performed for all study variables. We will calculate the mean, median, and standard deviation for quantitative variables, and the absolute and relative frequency for qualitative variables. We will use SPSS 19.0 (SPSS Inc., Chicago, Illinois, United States) to analyze the data statistically. An intention-to-treat analysis will be applied using the last observation carried forward method for missing data. Analysis of covariance with the baseline as the covariate will be used to assess differences in treatment outcomes between the two groups at each of the time points. To correct for an inflated risk of type 1 errors, multiple comparison procedures suggested by Ludbrook et al. [14] will be used for the outcome measure by using it as a covariate in the statistical analysis. A data safety monitoring board has been established to assess the progress of the trial, particularly the safety endpoints.

## Discussion

Despite advances in treatments with antimicrobial agents, mortality among hospitalized patients with SCAP is still high. SCAP remains the leading cause of death in the world [15]. TCM have been used for thousands of years in China for treating pneumonia, and it is widely used in many countries. Evidence from both clinicians and patients suggests that TCM may improve SCAP survival rates. However, the available evidence has been derived from only a few small randomized controlled trials.

Some studies have shown that TCM may have a beneficial effect on patients with CAP, including those with severe pneumonia [16]. In these patients, reviews show that TCM may reduce either length of hospital stay or mortality without causing complications [17]. However, insufficient data on SCAP are available, and the treatment in some studies was not according to syndrome differentiation. In view of these findings, a large prospective and adequately powered trial should conclusively determine the risks and benefits of adding TCM according to syndrome differentiation to the treatment of patients with SCAP.

In the current trial, we will include SCAP (risk class V in the pneumonia severity index) requiring hospitalization. According to previous studies, our study may not be powered to show a survival benefit. Our primary endpoint will be the rate of treatment failure, which includes treatment failure that occurred early or late. These criteria have been used previously with modifications.

Our secondary endpoints will be time to clinical stability, as defined by the guidelines of the Infectious Diseases Society of America and the American Thoracic Society, as well as length of intensive care unit and hospital stays, and in-hospital mortality.

In our study, we will administer an oral TCM granule for 28 days. All patients will be treated with antibiotics according to international guidelines. The treatment group will receive TCM according to the TCM syndrome. The control group will receive TCM placebo according to the TCM syndrome. Because a variety of treatment options have been adopted, we use the single blind method. We opted for an oral formula, since by choosing a uniform application formula, further bias can be prevented. Furthermore, the use of the placebo ensures the blinded design of this trial. Strict randomization will be implemented to minimize selection and evaluation bias.

TCM treatment is according to syndrome differentiation: *qing-fei jie-du hua-tan* (clearing away lung heat and detoxicating the phlegm) granule (Sanjiu Medical & Pharmaceutical Co., Ltd.) for phlegm-heat obstruction in the lung; *zao-shi hua-tan xie-fei* (eliminating dampness and eliminating phlegm) granule for pulmonary stagnation of phlegm; *qing-xin kai-qiao* (waking up the patient from unconsciousness by clearing away heart fire) granule for pathogenic heat trapped in the pericardium; *shenmai* injection (Sichuan Chuanda West China Pharmaceutical Co., Ltd.; 15 ml per ampulla, 10–60 ml each time when needed) for yin exhausted syndrome; and *shenfu* injection (Sanjiu Medical & Pharmaceutical Co., Ltd.; 10 ml per ampulla, 50–100 ml each time when needed) for yang exhausted syndrome.

In conclusion, this current large and adequately powered randomized trial will determine the risks and benefits of adding TCM for 28 days to the treatment of hospitalized patients with SCAP. Our hypothesis is that TCM treatment will result in a reduced rate of treatment failure in SCAP compared to conventional treatment.

### Study limitations

The results of this trial may be valid only for the specific type, dose, and duration of TCM studied. This study is powered for rate of treatment failure, but not for mortality; more patients would be needed for it to be powered to detect a clinically relevant difference in mortality.

### Trial status

At the time of manuscript submission, we have recruited 16 patients. The study will continue to enroll patients until 31 December 2019.

## Additional file

Additional file 1: SPIRIT 2013 checklist. (DOC 167 kb)

## Abbreviations

CAP: Community-acquired pneumonia
CAT: Chronic obstructive pulmonary disease assessment test
GOLD: Global Initiative for Chronic Obstructive Lung Disease
PSI: Pneumonia severity index
SCAP: Severe community-acquired pneumonia
SOFA: Sequential organ failure assessment
TCM: Traditional Chinese medicine
